# Inhibition of corticosterone synthesis impairs cued water maze consolidation, but it does not affect the expression of BDNF, CK2 and SGK1 genes in dorsal striatum

**DOI:** 10.3389/fnbeh.2024.1341883

**Published:** 2024-02-26

**Authors:** Rogelio Pegueros-Maldonado, Santiago M. Pech-Pool, Jaisson J. Blancas, Roberto A. Prado-Alcalá, Carlos Arámburo, Maricela Luna, Gina L. Quirarte

**Affiliations:** ^1^Departamento de Neurobiología Conductual y Cognitiva, Instituto de Neurobiología, Universidad Nacional Autónoma de México, Querétaro, Mexico; ^2^Departamento de Neurobiología Celular y Molecular, Instituto de Neurobiología, Universidad Nacional Autónoma de México, Querétaro, Mexico

**Keywords:** cued Morris water maze, corticosterone, glucocorticoid receptor, dorsal striatum, memory consolidation

## Abstract

Corticosterone (CORT) release during learning experiences is associated with strong memories and activity of the glucocorticoid receptor. It has been shown that lesions of the dorsal striatum (DS) of rats trained in the cued version of the Morris water maze impair memory, and that local injection of CORT improves its performance, suggesting that DS activity is involved in procedural memory which may be modulated by CORT. We trained rats in cued Morris water maze and analyzed the effect of CORT synthesis inhibition on performance, CORT levels, expression of plasticity-involved genes, such as the brain derived neurotrophic factor (BDNF), casein kinase 2 (CK2), and the serum/glucocorticoid regulated kinase 1 (SGK1), as well as the presence of phosphorylated nuclear glucocorticoid receptor in serine 232 (pGR-S232) in the DS. The inhibition of CORT synthesis by metyrapone reduced CORT levels in plasma, prevented its increment in DS and impaired the performance of cued water maze. Additionally, there was an increase of CK2 and SGK1 mRNAs expression in trained subjects, which was unrelated to CORT levels. Finally, we did not observe changes in nuclear pGR-S232 in any condition. Our findings agree with evidence demonstrating that decreasing CORT levels hinders acquisition and consolidation of the spatial version of the Morris water maze; these novel findings broaden our knowledge about the involvement of the DS in the mechanisms underlying procedural memory.

## 1 Introduction

During stressful experiences, glucocorticoid hormones such as corticosterone (CORT) in rodents and cortisol in humans are released into the bloodstream and have been associated with memory functions. It has been shown that CORT administration enhances memory formation in aversive tasks such as the Morris water maze (Sandi et al., 1997) and fear conditioning (Hui et al., 2004).

Evidence shows that CORT modulates memory processes through the activation of glucocorticoid receptors (GRs) (Medina et al., 2007). GRs are nuclear receptors which act as transcription factors to enhance or repress the expression of genes that are related to neural plasticity, namely the brain derived neurotrophic factor (BDNF) (Chen et al., 2017) that is related to calcium influx associated with glutamate signaling (Amaral and Pozzo-Miller, 2007); casein kinase 2 (CK2) (Datson et al., 2001), that is associated with NMDA receptor regulation (Kimura and Matsuki, 2008); and the serum/glucocorticoid regulated kinase 1 (SGK1) (Hinds et al., 2017), related to the expression of AMPA receptor subunits (Strutz-Seebohm et al., 2005).

This function is modulated by post-translational modifications, mainly by phosphorylation in serine residues; particularly, pGR-S232 has been shown to promote nuclear translocation (Wang et al., 2002). Most of the information about how glucocorticoids modulate memory has derived from studies on the explicit memory system where the hippocampus is its key element. However, the implicit or procedural memory system has been less studied. Procedural memory is related to the dorsal striatum (DS), which is involved in motor learning and stimulus–response behavior (Rice et al., 2015). Cued (procedural) Morris water maze learning has been shown to be modulated by CORT, since systemic administration enhances memory formation (Goodman et al., 2015). Lesions on the DS cause impairment on this task (Miyoshi et al., 2012), whereas local CORT injection enhances memory consolidation (Quirarte et al., 2009). Although CORT administration into DS modulates memory processes, the cellular mechanisms involved are yet to be explored. We hypothesized that cued Morris water maze performance could be related to an increase in plasma and striatal CORT levels as well as to the modulation of DS expression of BDNF, CK2, and SGK1 genes. We evaluated the effects of the inhibition of CORT synthesis by metyrapone on the performance of the cued water maze, CORT levels, gene expression and the nuclear presence of pGR-S232.

## 2 Method

A total of 98 male adult Wistar rats (*Rattus norvegicus albinus*, weighing between 250 and 350 g) were used in this study. They were obtained from the Animal Services Unit at the Instituto de Neurobiología, and maintained in the vivarium of our laboratory in individual acrylic home cages (24 × 21 × 45 cm) with access to food and water *ad libitum*, with a 12/12 h light/dark cycle, starting at 7:00 am. Animals were maintained in accordance with NORMA Oficial Mexicana NOM-062-ZOO-1999 (2001), and the recommendations of the Guide for Care and Use of Laboratory Animals of the National Research Council (2011). The protocols for these experiments were approved by the Ethics Committee of Instituto de Neurobiología. All behavioral observations were carried out between 8:00 h and 14:00 h, and the rats were randomly assigned to each group.

Metyrapone (Sigma-Aldrich, Germany, #M2696) or vehicle (VEH group) was injected subcutaneously 1.5 h before training. Metyrapone was diluted in 40% polyethylene glycol mixed with saline solution. The doses used were 7.5 (MET 7.5 group), 50 (MET 50 group), and 75 (MET 75 group) mg/Kg (Akirav et al., 2004). Dilutions were prepared at 0.01, 0.067, and 0.1 M respectively, to set a reference volume of 1.16 mL for all doses. Animals were weighed on training day to calculate the proportional injection volume.

The rats were trained in a water maze consisting of a black circular plastic tank (1.54 m in diameter and 0.60 m in height) filled with water (25 ± 1°C) to a depth of 21 cm, surrounded by black curtains to avoid the presence of distal cues. Four starting positions were equally spaced around the pool perimeter, dividing it into four quadrants. The rat was placed in the tank at one of the four designated starting points, facing the wall, and allowed to escape onto a visible platform (12 × 12 cm), marked by a white and green striped cylinder. If subjects missed the platform in the first trial, they were guided to it. After mounting the platform, the rat remained there for 10 s and was then placed in a holding box for 30 s until the start of the next trial. A total of eight trials were given and the platform was moved to a different location on each trial, such that each of the four quadrants contained the escape platform twice. The locations of the starting points were arranged such that distance to the escape platform (i.e., proximal or distal) and location of the platform relative to the starting point (i.e., left or right) were counterbalanced across trials. 48 h after the training session, each rat was given one retention trial. Escape latencies were measured with Any-maze software (Stoelting Co, United States).

For DS analyses protocols, rats were injected with VEH or metyrapone (75 mg/Kg) and trained as described. Three experiments were performed, where subjects were euthanized at different intervals after training to measure: (a) CORT levels in plasma and DS (15 min), (b) pGR-S232 in DS (1.5 h), and (c) DS mRNA expression (2.0 h); a control group that stayed in its home-cage until tissue collection -denominated CAGED- was added to each of these experiments. CORT levels were measured in plasma and DS homogenates with an ELISA CORT kit following the manufacturer’s instructions (Abcam, United States, #ab108821). We obtained trunk blood and froze the brains with an isopentane solution tempered with dry ice. Blood was centrifuged at 3000 g for 10 min to obtain the plasma. Using a stainless-steel matrix, 2 mm-thick sections that included DS were obtained (0.0 to 2.0 mm from bregma) (Paxinos and Watson, 2007), which were then dissected, weighed, and mixed with Kit diluent buffer, followed by centrifugation at 5000 g for 15 min, taking the supernatant for measurements. The obtained data was referred to tissue weight to express results in ng/mg.

For the assessment of mRNA expression, total RNA was extracted from ~25 mg of DS. We used the Direct-zol RNA MiniPrep Plus (Zymo Research, United States) kit according to the manufacturer’s instructions. cDNA was synthesized by reverse-transcription using 1 μg of total RNA. Reverse transcriptase (M-MLV Reverse transcriptase, Promega, 200 U/μL) reaction was performed following previous studies (Pech-Pool et al., 2020). BDNF, CK2 and SGK1 mRNA expression was measured by real time PCR (qPCR) in a StepOne Thermocycler Real-Time PCR system (Applied Biosystems) while using Maxima SYBR Green qPCR Master Mix (2X) (ThermoFisher Scientific, United States). We used 0.5 μM of each specific primer (Supplementary Table S1) and the following cDNA dilutions: 1:5 for BDNF; 1:5 for CK2; 1:10 for SGK1; and 1:10 for 18S. The reactions were performed as follows initial denaturation at 95°C for 10 min, followed by 45 cycles of 95°C for 15 s, 60°C for 30s and finally 75°C for 30s. The relative content of mRNAs was calculated with the comparative threshold cycle (Ct) method using the 2^-–ΔΔCT^ formula, where gene expression was relative to the genomic mean of 18S mRNA (Livak and Schmittgen, 2001).

We also semi-quantified total GR (1:2000; Santa Cruz Biotech, United States, #sc-8992) and GR phosphorylated on serine 232 (pGR-S232) (1,1,000; Cell Signaling Technology, United States, #4161) in a nuclear fraction of the DS. Samples were homogenized with 120 μL lysis buffer (Abcam, United States, #ab152163) containing a COMPLETE protease inhibitor cocktail (Roche #11836153001) and Halt phosphatase inhibitor (ThermoFisher Scientific, United States, #78420), then centrifuged at 1100 g for 15 min to obtain a pellet with the nuclear fraction (Moutsatsou et al., 2001).

SDS-PAGE and Western blot were performed as previously described (Ponce-Lina et al., 2020). Membranes were incubated with a goat anti-rabbit secondary antibody conjugated to horseradish peroxidase (1:3,000; Invitrogen, United States, #ab6721) for 2 h at room temperature. Immunoreactive bands were developed by chemiluminescence using ECL (Amersham Biosciences) on hyperfilm (Amersham Biosciences). Luminograms were analyzed by densitometry using the Image Lab software (Bio-Rad). The stripping method was used to normalize GR and p-GRS232 immunoreactivity with the loading protein for nuclear fraction Lamin A/C (1:5,000, Sigma-Aldrich, United States, #SAB4200236) as reported elsewhere (Mao et al., 2013).

For statistical analyses, training escape latencies were analyzed with a two-way ANOVA and the Tukey *post hoc* test for treatment effects across trials. Retention test escape latencies, CORT and mRNA measures were analyzed with a one-way ANOVA and Tukey *post hoc* tests. Western blot results were analyzed with a one-sample Wilcoxon signed rank test. A *p* < 0.05 value was considered statistically significant. Data were analyzed with GraphPad Prism software.

## 3 Results

**Figure 1:**
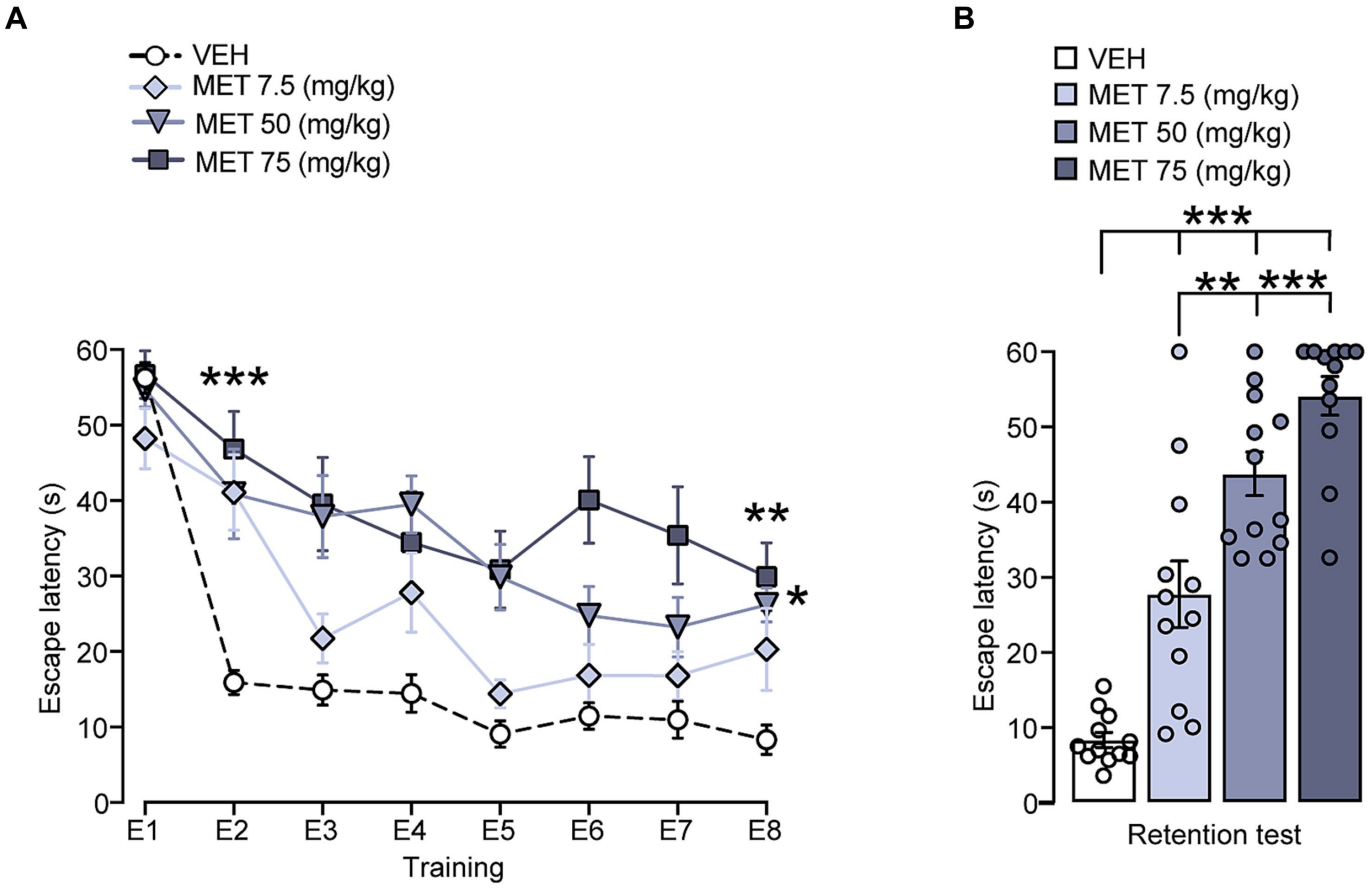
Effect of CORT synthesis inhibition on cued Morris water maze training and retention performance. **(A)** There were significant differences between VEH and all metyrapone treated groups in the second training trial; VEH was also different from MET 50 and MET 75 in the last trial. *** *p* < 0.001, ** *p* < 0.01, * *p* < 0.05. **(B)** On the retention test MET 75 displayed longer escape latencies than the other groups. *** *p* < 0.001, ** *p* < 0.01. *n* = 12 for all groups. Data represent mean ± S.E.M. In this figure and in the following figures, the small circles represent individual values.

Initially, we assessed the impact of metyrapone on performance in the cued Morris water maze. Within group comparisons for training performance (Figure 1A) showed statistical differences in the trial factor [*F* (7, 352) = 29.32, *p* < 0.001], the treatment factor [*F* (3, 352) = 42.31, *p* < 0.001], and the group x trial interaction [*F* (21, 352) = 29.32, *p* = 0.019], which suggests that lowering CORT levels exerts a detrimental effect on learning, as seen in the second trial, where we found differences when comparing VEH against MET 7.5 (*p* = 0.001), MET 50 (*p* < 0.001), MET 75 (*p* < 0.001), and in the last trial, against MET 50 (*p* = 0.013) and MET 75 (*p* = 0.001). The treatment effect was more evident in the retention test [*F* (3, 44) = 44.88, *p* < 0.001] where we found impairment in all metyrapone-treated groups when comparing VEH against MET 7.5 (p < 0.001), MET 50 (*p* < 0.001), MET 75 (*p* < 0.001); MET 7.5 also differed from MET 50 (*p* = 0.002), and MET 75 (*p* < 0.001) (Figure 1B). The velocity data did not yield significant differences among the groups (data not shown), which suggests that there was no motor impairment.

**Figure 2:**
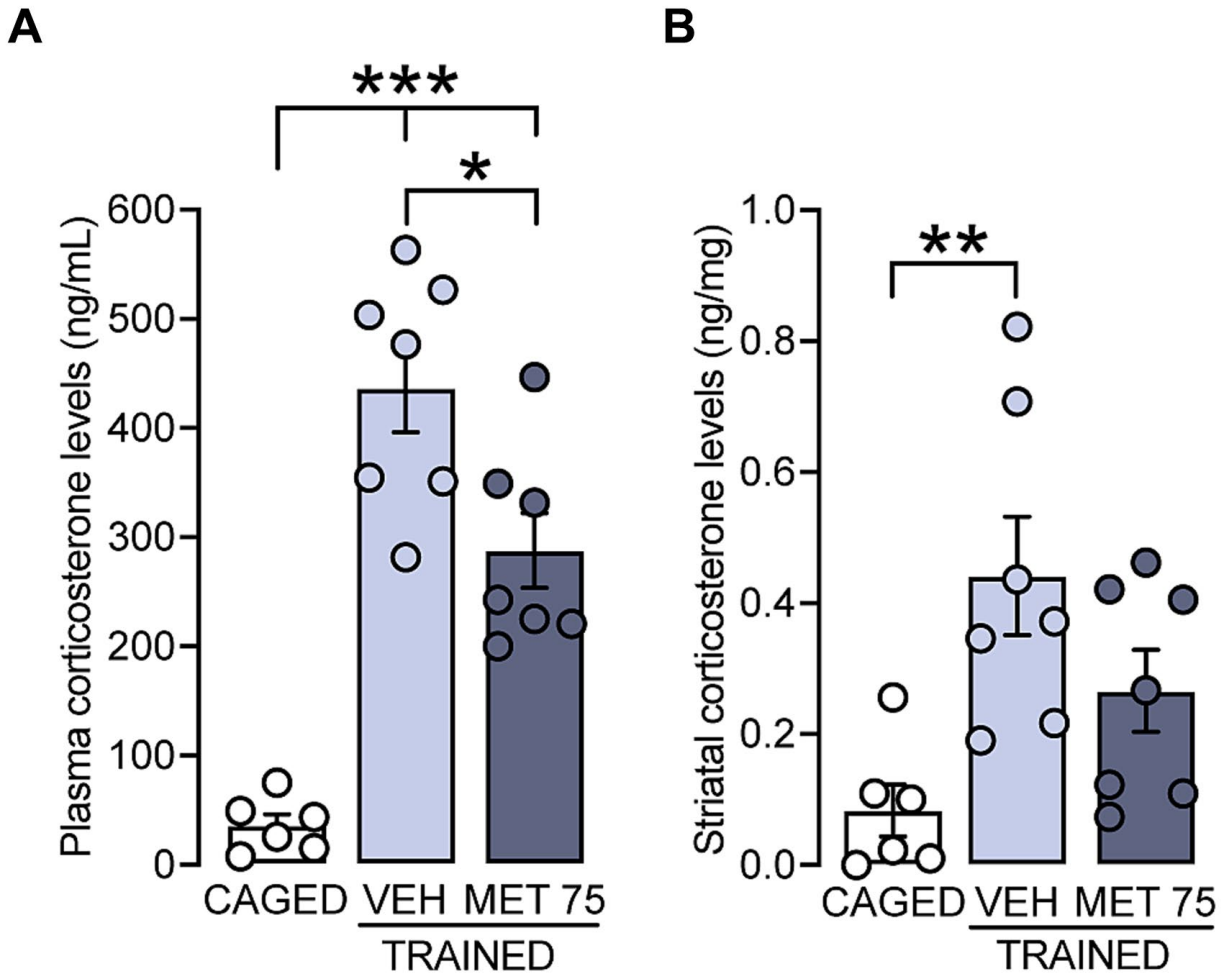
CORT levels in rats trained in the cued water maze. Plasma and DS samples were collected 15 min after training. **(A)** CORT levels in plasma. The VEH and MET 75 groups had higher CORT levels than the CAGED group, and the MET 75 group showed lower CORT levels than VEH, ***p < 0.001, *p < 0.05. **(B)** Striatal levels of CORT. There was a significant increase in CORT level only in VEH, as compared to the CAGED group, ** *p* < 0.01. *n* = 6–7 rats per group. Data represent mean ± S.E.M.

Secondly, we measured CORT levels in plasma and DS after treatment with metyrapone. There was a significant effect of metyrapone on plasmatic CORT levels [*F* (2, 17) = 37, *p* < 0.001]; the VEH and MET 75 groups had higher levels than the CAGED group (*p* < 0.001 for both comparisons), and the MET 75 group showed lower CORT levels than VEH (*p* = 0.011) (Figure 2A). In the case of DS, there were also statistical differences among the groups [*F* (2, 17) = 6.363, *p* = 0.008] with a significant increase of CORT in VEH (*p* = 0.006) but not in MET 75 (Figure 2B). Overall, these results show that metyrapone reduces CORT plasma elevation and prevented the DS increase provoked by cued Morris water maze training.

**Figure 3:**
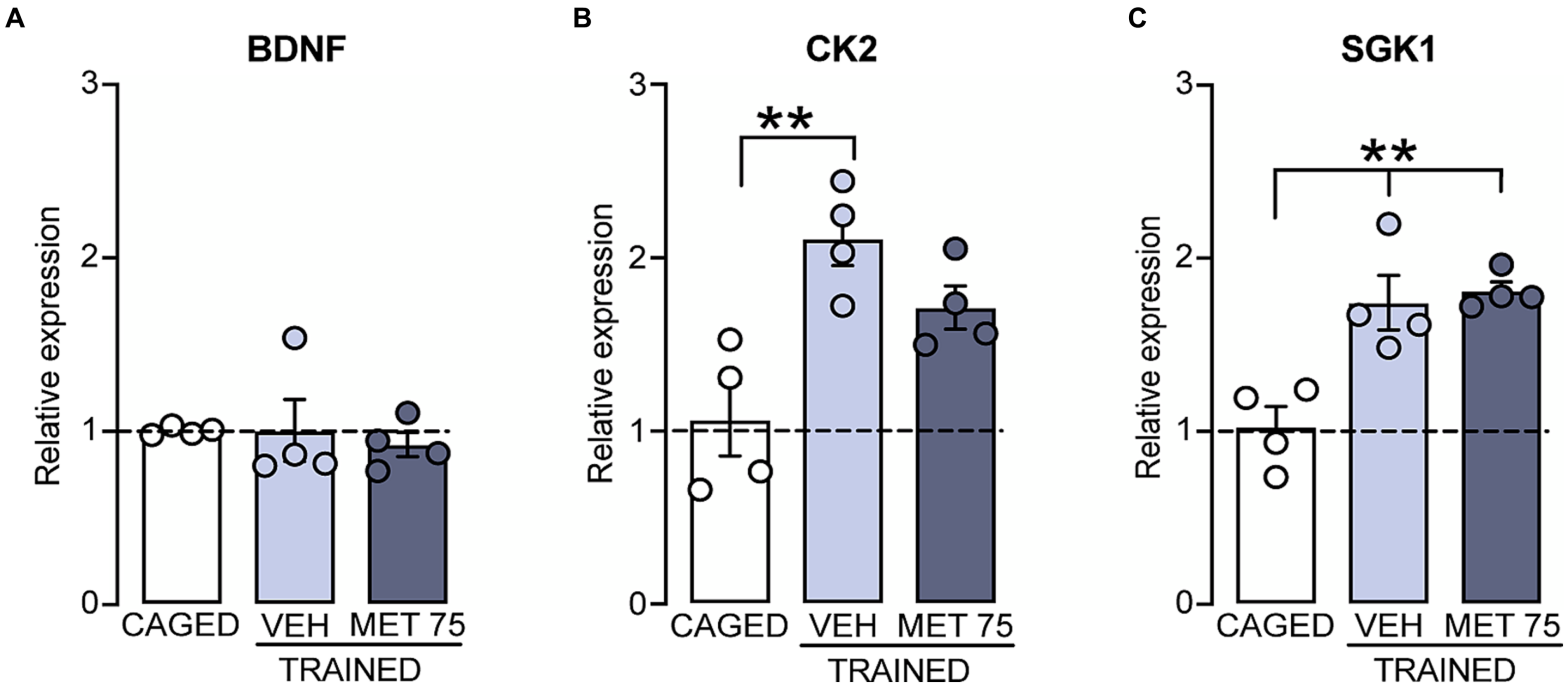
Striatal mRNA expression of BDNF, CK2, and SGK1 relative to housekeeping gene 18S, in rats trained in the cued Morris water maze. DS tissue was collected 2.0 h after training. **(A)** There were no significant differences among the groups regarding BDNF mRNA. **(B)** VEH and MET 75 had a higher expression of CK2 mRNA than CAGED; **p < 0.01. **(C)** VEH and MET 75 had a higher expression of SGK1 mRNA than CAGED (*p* < 0.01 for both comparisons); **p < 0.01. *n* = 4 for all groups. Data represents mean ± S.E.M and were calculated as a percentage of the relative expression value of the CAGED group.

We then quantified the expression levels of BDNF, CK2 and SGK1 mRNAs (Figure 3). We found no treatment effect on BNDF expression [*F* (2, 9) = 0.1700, *p* = 0.846]. However, there was a significant treatment effect on the expression of CK2 [*F* (2, 9) = 10.05, *p* = 0.005]; the trained VEH group showed a higher expression than the CAGED group (*p* = 0.0041), and there was a clear tendency of increased expression in MET 75 (*p* < 0.053). By the same token, there was a significant treatment effect on the expression of SGK1 [*F* (2, 9) = 13.71, *p* = 0.001]; both VEH and MET 75 displayed a higher expression of SGK1 mRNA than CAGED (*p* < 0.004 and = 0.0028, respectively). There were no significant differences between the trained groups regarding the expression of any of the studied genes.

**Figure 4:**
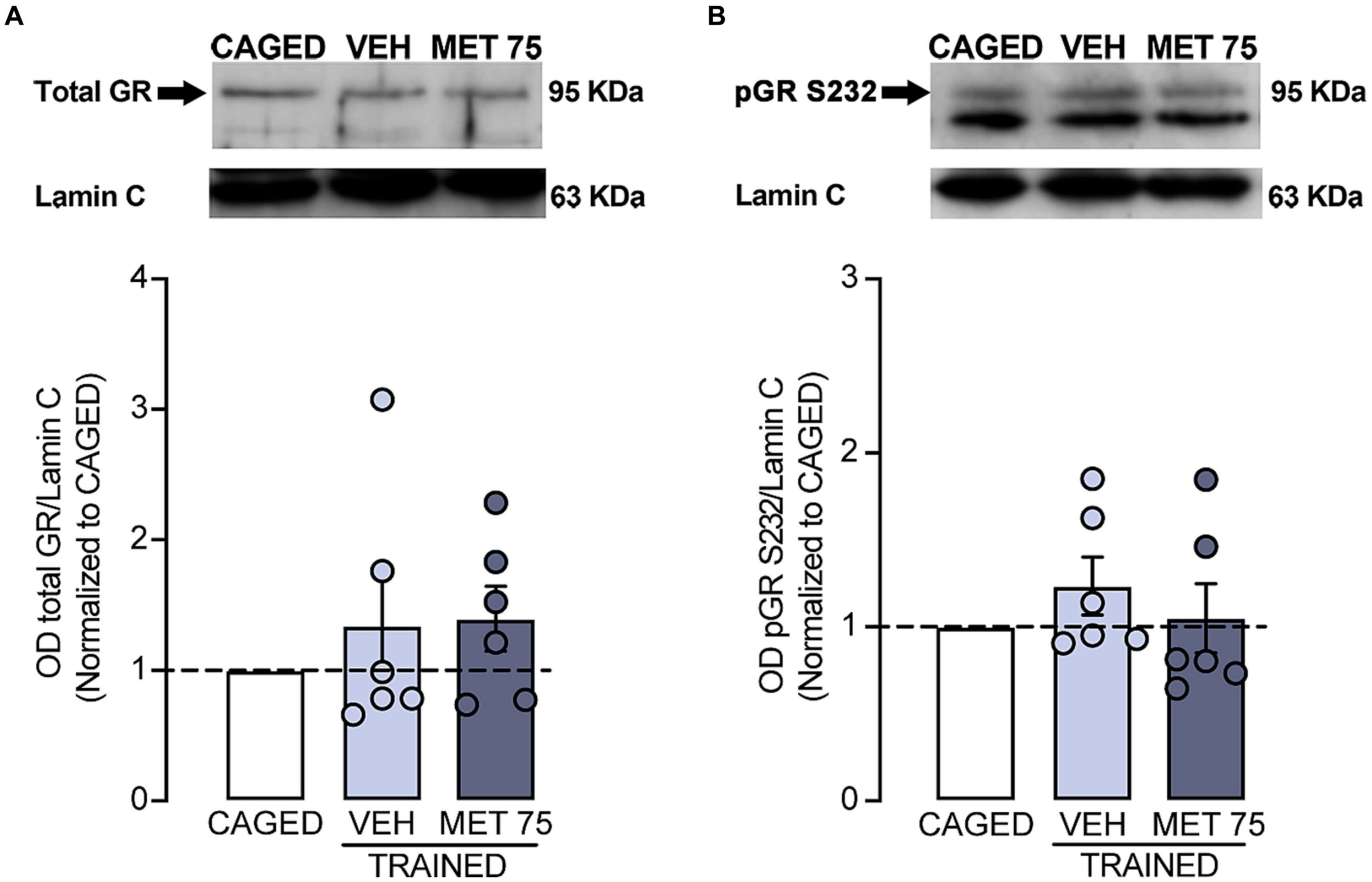
Striatal nuclear GR presence and p-GRS232. Lamin C was used as loading control. DS tissue was collected 1.5 h after training. **(A)** Representative blot and optical density of total GR levels. **(B)** Representative blot and optical density of p-GRS232. *n* = 6 for all groups. Data represents mean ± S.E.M. and were calculated as a fold change value of the CAGED group.

Lastly, we measured the presence of GR (Figure 4A) and pGR-S232 in DS (Figure 4B). We found no significant differences regarding total GR between CAGED and VEH (*Z* = 1 *p* > 0.999), nor between CAGED and MET 75 (*Z* = 11, *p* = 0.312); also, there were no significant differences in pGR-S232 between CAGED and VEH (*Z* = 1 *p* = 0.437), nor between CAGED and MET 75 (*Z* = 11, *p* > 0 0.999).

## 4 Discussion

Our behavioral results agree with evidence that systemic pretraining administration of metyrapone with similar doses, caused dose-dependent impairment in training and retention of the spatial version of the Morris water maze task (Akirav et al., 2004); this indicates a robust effect of corticosterone synthesis inhibition on both versions of this task. The cued Morris water maze promotes a stimulus–response strategy which involves DS activity (Miyoshi et al., 2012), structure that is modulated by CORT in this task (Quirarte et al., 2009). Our data suggests that decreasing CORT levels affects DS activity in this task.

We found that water maze training induced a significant increase in CORT levels both in plasma and DS; these results were expected because in this task swimming, by itself, is stressful (Harrison et al., 2009). In the case of DS, training induced an increased level of CORT, which was reversed by metyrapone, as evidenced by the lack of difference between the CAGED and MET 75 groups.

The results of gene expression showed an interesting pattern. BDNF did not show changes in its expression, while CK2 and SGK1 significantly increased in the DS of trained subjects, an effect that was independent of CORT levels; these results disagree with other findings of hippocampal expression of these genes (Datson et al., 2001; Chen et al., 2017; Hinds et al., 2017).

BDNF has shown a functional importance in neurotransmission modulation (particularly glutamate) and regulation of calcium intracellular elevations in the hippocampus (Amaral and Pozzo-Miller, 2007); we found no changes in BDNF expression in the DS of trained subjects, a result that is at variance with evidence that shows that BDNF is involved in learning and memory in the dorsal striatum (D'Amore et al., 2013). There are instances, however, where increased BDNF signaling impairs cognitive functions (Huang et al., 2021), and, as demonstrated by Albeck et al. (2005) there were no changes in striatal BDNF expression after an aversively motivated task. These discrepant results may indicate that depending on the behavioral paradigm being studied BDNF may be involved at different times after the learning experience, or that BDNF expression could be regulated by CORT in a manner that was not detected in our study.

On the other hand, CK2 and SGK1 increments may be related to DS plasticity and memory consolidation, as their manipulation has been shown to affect spatial Morris water maze performance (Tsai et al., 2002; Chao et al., 2007). In the case of SGK1, there is evidence that it regulates the AMPA receptor subunit expression (Strutz-Seebohm et al., 2005), which is important for long-term potentiation (LTP). Furthermore, CK2 has been also associated with LTP via the direct modulation of NMDA receptors (Kimura and Matsuki, 2008), and with dopamine mechanisms in the striatum, where a selective knockout affected dopamine receptor signaling and caused motor impairment (Rebholz et al., 2013). SGK1 and CK2 increase may facilitate the crosstalk of glutamate and dopamine signaling, which is required for striatal plasticity (Cerovic et al., 2013).

In our last experiment we intended to relate gene expression with GR nuclear presence; however, we did not find any statistical differences in our experimental conditions. GR has a moderate expression in the striatum (Morimoto et al., 1996) and it has been described that for GR to translocate into the nucleus, it requires high levels of CORT (Du et al., 2009). Previous measurements from our laboratory (Ponce-Lina et al., 2020) demonstrated that a high foot shock (1.5 mA) elevates systemic CORT to ~1,600 ng/mL. Here, we found a 476.76 ng/mL increase of CORT plasma levels. Considering these two factors, it is possible that for GR to modulate DS a high elevation of CORT is needed, as we have found previously, since moderate stress learning conditions did not induce pGR-S232 presence in the DS (Gonzalez-Franco et al., 2023).

It is important to consider that the dorsal striatum, being a heterogeneous structure with lateral and medial divisions, exhibits functional differences related to memory types. The absence of differences in gene expression and pGR-S232 may be due to the fact that the whole dorsal striatum was analyzed. Specifically, the dorsolateral region plays a role in cue task memory formation (Miyoshi et al., 2012), while the medial region influences the transition from spatial to procedural performance, mediated by glucocorticoids (Lozano et al., 2013). Future studies should explore the potential functional regionalization of memory systems and gene expression in the DS.

In summary, our findings agree with evidence showing that decreasing CORT levels impairs acquisition and consolidation of spatial Morris water maze learning since metyrapone treatment also affected the performance in the cued (procedural) version. Training produced an increase in DS CORT levels in VEH, and this effect was prevented in MET 75 subjects; this may suggest that the elevation of CORT in the DS is necessary for consolidation. We, however, did not find the same pattern in pGRS232 nuclear translocation, this may implicate other possible GR activation mechanisms in the DS.

Furthermore, we found an elevation in mRNA expression of CK2 and SGK1 that was independent of CORT levels, which suggests that these genes have other regulatory mechanisms that may open interesting research possibilities for procedural memory consolidation in the DS.

## Data availability statement

The original contributions presented in the study are included in the article/Supplementary material, further inquiries can be directed to the corresponding authors.

## Ethics statement

The animal study was approved by the Ethics Committee of Instituto de Neurobiología, UNAM. The study was conducted in accordance with the local legislation and institutional requirements.

## Author contributions

RP-M: Conceptualization, Formal analysis, Investigation, Methodology, Writing – original draft, Writing – review & editing. SP-P: Conceptualization, Formal analysis, Investigation, Methodology, Writing – original draft, Writing – review & editing. JB: Formal analysis, Investigation, Methodology, Writing – original draft. RP-A: Formal analysis, Investigation, Project administration, Resources, Visualization, Writing – original draft, Writing – review & editing. CA: Formal analysis, Project administration, Writing – original draft, Writing – review & editing, Funding acquisition, Investigation, Visualization. ML: Formal analysis, Funding acquisition, Project administration, Resources, Writing – original draft, Writing – review & editing, Conceptualization, Investigation, Methodology, Supervision, Validation, Visualization. GLQ: Conceptualization, Formal analysis, Investigation, Methodology, Project administration, Resources, Supervision, Validation, Visualization, Writing – original draft, Writing – review & editing.
