# Supplementary figures and images for: Inhibition of corticosterone synthesis impairs cued water maze consolidation, but it does not affect the expression of BDNF, CK2 and SGK1 genes in dorsal striatum
Source: Front Behav Neurosci. 2024 Feb 26;18:1341883. doi: 10.3389/fnbeh.2024.1341883 (PMC10925660; doi:10.3389/fnbeh.2024.1341883)

Table S1. Primers used for qPCR procedures.


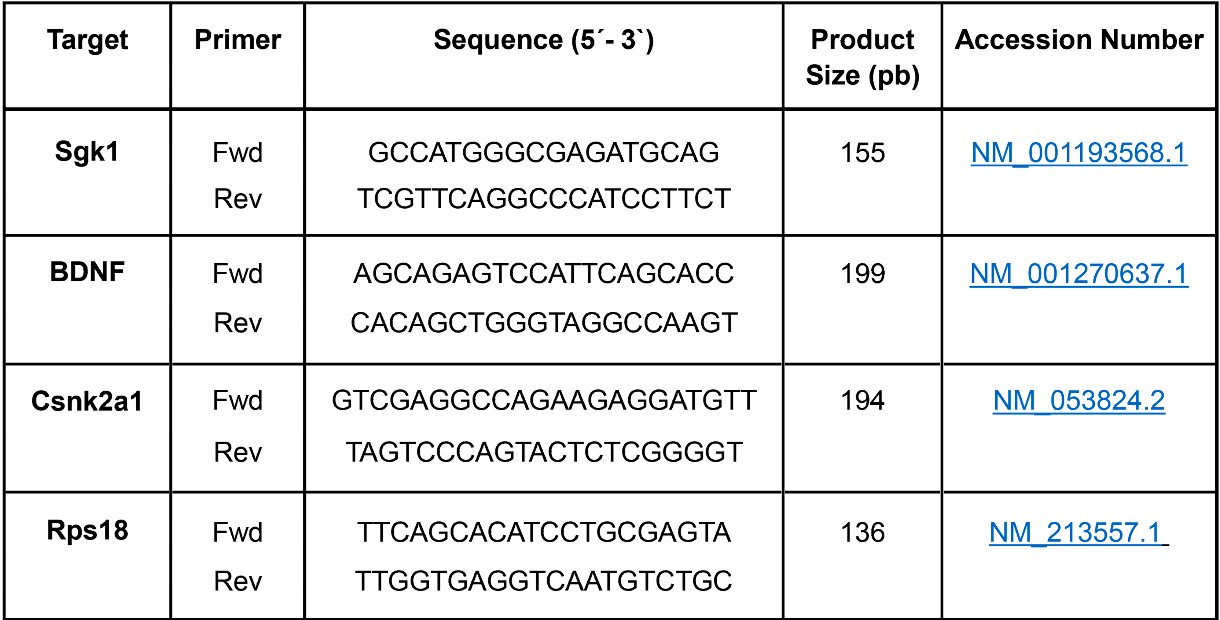

Supplement: Supplementary file 1 [file Table_1.DOCX]
